# Postoccupancy Evaluation of a New Hospital: The Relationship With Work Engagement

**DOI:** 10.1177/19375867251351026

**Published:** 2025-06-30

**Authors:** Hanna Petäjä, Pinja Krook, Suvi Kuha, Jouko Katajisto, Outi Kanste

**Affiliations:** 1 Research Unit of Health Sciences and Technology, Faculty of Medicine, 6370 University of Oulu, Oulu, Finland; 2 Department of Mathematics and Statistics, 8058 University of Turku, Turku, Finland; 3 577217 Medical Research Center Oulu, University Hospital and University of Oulu, Oulu, Finland

**Keywords:** hospital design and construction, built environment, physical work environment, work engagement, personnel turnover, evidence-based facility design

## Abstract

**Aim:** To assess staff satisfaction with the physical work environment (PWE) and its relationship with work engagement and turnover intention through a new hospital's postoccupancy evaluation (POE). **Background:** The healthcare workforce shortage has intensified globally. POE is a well-established method for collecting information on the success of the PWE. While strong work engagement is associated with lower turnover intention, research on their relationship with satisfaction in PWE in hospitals is limited. **Methods:** The study used a cross-sectional survey design. Data were collected at a public hospital in Finland from January to February 2024, using a POE questionnaire, the Utrecht Work Engagement Scale-3, and the Turnover Intention Scale. A total of 510 hospital staff members participated. The data were analyzed using correlations and multifactor analysis of variance. **Results:** Overall satisfaction with the physical work environment was relatively high. Satisfaction in security and safety, comfort, and architecture was strongly correlated with most other PWE categories. Physicians and hospital support and logistics staff were more satisfied with the PWE than nurses. A moderate relationship was found between satisfaction with the PWE and work engagement. The perception of comfort with the PWE and satisfaction with security and safety were moderately associated with work engagement. The relationship between satisfaction with the PWE and turnover intention was weak. **Conclusion:** The results suggest that when planning new hospitals, attention should be paid to developing the PWE, especially in terms of safety and security and comfort, since it may impact staff work engagement.

## Introduction

All countries are struggling with a continuous healthcare workforce shortage (WHO, 2023). Workforce demand is high and evolving due to aging populations, an aging healthcare workforce, rising disease burdens, and health emergencies (Agyeman-Manu et al., 2023). The situation is expected to worsen in the future as the World Health Organization estimates a shortfall of 10 million healthcare workers by 2030 (WHO, 2023).

Healthcare workforce availability has weakened globally and become even more concerning following the COVID-19 pandemic (WHO, 2022b). The WHO's Global Strategy on Human Resources for Health (2016) highlights that all countries should consider the importance of better working environments in their strategies and investments. In January 2024, health ministries from almost 50 countries signed a declaration to address healthcare workforce shortages by concerted action to improve the working conditions of healthcare workers (OECD, 2024).

The design of healthcare facilities has an enormous impact on efficiency and outcomes (Hicks et al., 2015; Kalantari & Snell, 2017). Good hospital design has been successful in shortening transfer distances, which promotes teamwork and informal communication and further improves profitability and quality (Karvonen et al., 2017). Approximately 75% to 80% of avoidable total costs of building a new facility are controllable at the design stage (Hicks et al., 2015). In addition, a good hospital design and physical working environment have helped to prevent medical errors, improve patient safety, and reduce costs (Hicks et al., 2015). The physical work environment is a part of the workplace facility that can be detected by human or electronic senses (WHO, 2010). These include structure, indoor air, furniture, machines, chemicals, materials, and processes that are present or occur in the workplace and can affect workers’ physical or mental safety, health, and wellbeing (Bentulila et al., 2024; Eijkelenboom & Bluyssen, 2022). Satisfaction with the physical work environment is a key indicator of both the performance and wellbeing of workers (Lupo et al., 2021). However, it is important to recognize that mismatches between design intentions and daily practices may arise when bricks (physical environment), bytes (technology), and behavior (work practices) are not fully aligned throughout the hospital design and implementation process (van Heel & van Oel, 2022).

Postoccupancy evaluation (POE) is a well-established method for collecting information on the success of the physical work environment. POE assesses how well buildings match users’ needs and obtains feedback on the building's performance in use (Aalto et al., 2017; Li et al., 2018). It is a way to measure the effectiveness of new architectural designs in achieving their intended purpose (Kalantari & Snell, 2017) and aims to identify ways to improve building design and performance for purpose as well as generalize lessons learned for a broader application to help design teams and owners make more informed decisions in future projects (Aalto et al., 2017; Li et al., 2018). Previous POE studies have focused mainly on patient health and wellbeing (Jouppila, 2022), while recently, there has also been interest in staff health and performance (Brambilla et al., 2019; Li et al., 2018). Focusing on staff experiences of working conditions is vital to enhancing professionals’ work engagement (Slåtten et al., 2022). Recent evidence also highlights the importance of the timing of POE. Pruijsten et al. (2024) demonstrated that staff perceptions of a newly built hospital environment evolved between 1 and 2 years after relocation, suggesting that early evaluations may not fully capture staff habituation and adaptation to new workflows and physical layouts.

Work engagement is a work-related positive, enriching emotional, and cognitive status, which comprises vigor (i.e., high levels of psychological energy during work), dedication (i.e., a sense of significance and challenge regarding work), and absorption (i.e., total immersion in one's work) (Schaufeli & Bakker, 2004a). Studies demonstrate that high work engagement is associated with increased job satisfaction, enhanced work performance and care quality, positively impacted healthcare systems, and reduced turnover intention (Moloney et al., 2018; Slåtten et al., 2022; Wang et al., 2023).

Turnover intention reflects the probability and willingness that an employee will leave an organization and look for a new position in either the same or different professions (Lu et al., 2023; Poon et al., 2022). It may not always lead to actual turnover, but it is found to be a reliable variable to forecast turnover behavior. The stronger the turnover intention, the more likely employees will act in a turnover-like manner (Lu et al., 2023). High turnover rates cause challenges in staffing healthcare facilities adequately, which has several implications for the quality of care. Low staffing is associated with, for example, increased patient mortality rates and a lower quality of care (Poon et al., 2022; Vardaman et al., 2014).

In previous studies, the physical work environment has been found to influence the intention to leave (Khan et al., 2019; Sadler et al., 2011) and predict work engagement (Hakanen et al., 2005) among healthcare professionals. Additionally, some preoccupancy evaluation and POE studies of an old and new hospital indicated that satisfaction in the new hospital increased. Even so, the staff's intention to quit did not vary (Alvaro et al., 2016), and their commitment to the organization did not improve (Schreuder et al., 2015).

However, there are currently no POE studies regarding work engagement and turnover intention in healthcare settings focusing on new hospitals. Therefore, our study aimed to assess staff satisfaction with the physical work environment and its relationship to work engagement and turnover intention through the POE of a new hospital.

## Methods

### Study Design

This study employed a descriptive cross-sectional survey design (Polit & Beck, 2022). Guidelines for STrengthening the Reporting of OBservational studies in Epidemiology (STROBE) were followed to improve the quality of reporting observations in the study (von Elm et al., 2008).

### Data Collection and Participant Recruitment

The data was collected using an online survey (Webropol) among hospital staff in a new public hospital in Finland from January to February 2024. The new hospital (180 hospital beds, ca. 55,000 m^2^) had been used for under 2 years. The hospital provides specialized medical care for child and adolescent diseases, childbirth and women's disorders, otolaryngology conditions, and mouth and jaw diseases. It also provides imaging services.

A target organization was selected based on convenience sampling (Polit & Beck, 2022). An online survey was emailed to all 1,542 hospital staff members through contact persons in different hospital units. The inclusion criterion was that the participant had to work at the target hospital during the survey.

To ensure that professionals had the opportunity to participate in the study, the questionnaire was kept as compact as possible, and completing it required about 15 to 20 min. To improve response activity, the hospital staff received two email reminders to complete the survey every week. The survey was closed after 3 weeks.

### Ethical Considerations

Research permission was obtained from the local Clinical Research Center. The study did not require ethics committee approval under the Finnish Medical Research Act 488/1999 (Vic), as it did not involve minors, direct or indirect physical or physiological harm to the participants, or clinical trials, and responding to the questionnaire did not require actions that would impact respondents’ wellbeing. European Union data protection legislation, such as the General Data Protection Regulation 2016/679, was adhered to in collecting and processing personal data (European Union, 2016).

The hospital's senior management was contacted before the study to obtain approval for staff to answer the questionnaire during working hours. The hospital staff was informed about the study by an email outlining its purpose, data collection, data preservation details and subsequent use, the voluntariness of participation, and the anonymity and contact information of the researchers. Respondents were informed that by answering the survey, they gave informed consent to participate in the study. Researchers had no access to staff information as the questionnaire was sent via contact persons in the hospital's units. Survey participants were given the opportunity to enter a voluntary draw through a separate survey, where gift cards for lunch restaurants and a grocery store were randomly awarded.

Our study was carried out and documented in line with the Finnish National Board on Research Integrity guidelines (Finnish National Board on Research Integrity, 2023). The ethical principles of the World Medical Association and the Declaration of Helsinki were followed in all study phases (World Medical Association, 2013).

### Measures

The questionnaire contained four sections: participants’ characteristics, POE survey, work engagement scale, and turnover intention scale. The participants’ characteristics included gender, age, professional status, current work experience, healthcare work experience, and type of employment contract (Table 1).

**Table 1.** Participants’ Characteristics (*n* = 510).

Variable	*n*	%
Gender		
Women	459	90
Men	47	9.2
Other	4	0.8
Professional status		
Nurses	281	55.1
Physicians	63	12.4
Other healthcare professionals*	71	13.9
Nonclinical staff**	53	10.4
Hospital support and logistics staff	42	8.2
Type of contract		
Permanent	409	80.2
Temporary	101	19.8
Work experience in current work		
< 6 months	30	5.9
6 months–4 years	149	29.2
5–10 years	111	21.8
11–20 years	143	28
Over 21 years	77	15.1
Professional experience in healthcare		
< 5 years	79	15.5
5–10 years	95	18.6
11–20 years	152	29.8
Over 21 years	184	36.1

	Mean	SD (range)
Age (years)	43.6	11.4 (19–67)

* Audiologist, occupational therapist, physiotherapist, psychologist, social worker, speech therapist, and ward pharmacist.

** Coordinator, secretary, ward manager, and manager.

SD=standard deviation.

The satisfaction with the physical work environment was explored using the POE questionnaire, which was tested and modified in connection with the user-oriented hospital project (Jouppila, 2022; Yli-Karhu, 2015) in another region. The measurement included 116 items in 13 main categories: hospital yards and entrances, architecture, acoustics, lighting, durability, functionality, indoor conditions, safety and security (structural, dangerous situations, operational, and systems), privacy, social interaction, comfort (patient and staff), accessibility, and usability. A five-point Likert scale was used (1 = completely disagree, 2 = somewhat disagree, 3 = somewhat agree, 4 = completely agree, 0 = does not concern me). The preferred mean for POE categories was 2.5 on a scale of 1 to 4, indicating that half of the respondents fully or mostly agree (Jouppila, 2022). Permission to use the POE was obtained from the developer of the questionnaire, and it was used in its original form to ensure comprehensive coverage of the physical work environment across various staff roles in the new hospital.

Work engagement was explored through an Ultra-Short Measure for Work Engagement (UWES-3), which consists of three items, each representing one particular aspect of work engagement: vigor, dedication, and absorption (Schaufeli et al., 2019). A seven-point Likert scale was used (0 = never, 1 = a few times a year, 2 = once a month, 3 = a few times a month, 4 = once a week, 5 = a few times a week, and 6 = daily). The three-item UWES has proven to be a reliable and valid indicator of work engagement (Schaufeli et al., 2019). The interpretation of the average values for work engagement is as follows: an average score below 1.44 is considered very low, between 1.44 and 3.43 is considered low, between 3.44 and 4.53 is considered moderate, between 4.54 and 5.30 is considered above average, and scores above 5.30 are considered high (Hakanen, 2009). The UWES-3 has already been translated into Finnish (Hakanen, 2009) and is freely available for noncommercial scientific research purposes (Schaufeli & Bakker, 2004b).

Turnover intention was measured using a three-item Turnover Intention Scale (TIS) created by Michaels and Spector (1982). A six-point Likert scale was used (1 = strongly disagree, 2 = moderately disagree, 3 = slightly disagree, 4 = slightly agree, 5 = moderately agree, and 6 = strongly agree; Spector, 2023b). TIS has proven to be a reliable instrument to measure turnover intention (Bothma & Roodt, 2013). The questionnaire was translated from English into Finnish using forward translation and expert panel back-translation (WHO, 2022a). TIS is free to use for noncommercial research (Spector, 2023a).

### Statistical Analysis

The data was analyzed using IBM SPSS Statistics 29.0 software (IBM Corp., 2023). Descriptive statistics, including frequencies, percentages, mean, standard deviation, and range, were utilized to summarize the background information. The sum variables were formed from the categories of the POE questionnaire, UWES-3, and Turnover Intention Scale. In the POE questionnaire, all questions were mandatory; however, respondents could select “does not concern me” for items they felt were not applicable. These responses were excluded from the analysis.

The correlation between the POE questionnaire and the sum variables formed by POE categories, UWES-3, and TIS were examined using Spearman's rho. The correlation between variables was considered weak if the absolute value of the correlation coefficient was <0.3. The correlation was considered moderate if it ranged between 0.3 and 0.5 and strong if it exceeded 0.5.

Multifactor analysis of variance was used to find the effects of background factors on the sum variables. The main effect model was used, in which continuous variables were utilized as covariates and categorical variables were utilized as fixed factors. Sidak adjustments for multiple comparisons were used for pairwise comparisons. The sample size (central limit theorem) was large enough to use parametric tests without concerns about normality assumptions. The statistical test was considered significant if the *p*-value was ≤ .05 (Polit & Beck, 2022).

## Results

### Background Information of Participants

A total of 510 respondents participated, yielding a response rate of 33%. The majority were female, and the mean age was 43.6 years. Over half of the respondents were nurses; just under a fifth worked in administration or support services and logistics. Four-fifths of the respondents have permanent contracts, and more than half had more than 11 years of experience working in healthcare (Table 1).

### Internal Consistency of the Survey Scales

The internal consistency of the scales was assessed using Cronbach's alpha coefficient. It is deemed acceptable if Cronbach's alpha levels are measured at 0.7 or higher (Boateng et al., 2018). The alpha values of the overall measures were good for turnover intention (0.90) and work engagement (0.80). The values in POE categories ranged from 0.70 to 0.92, the alpha value for the entire POE being 0.99. Descriptive statistics and Cronbach's alphas of POE, work engagement, and turnover intention are presented in Table 2.

**Table 2.** Descriptive Statistics and Cronbach's Alphas of Postoccupancy Evaluation, Work Engagement and Turnover Intention.

Category	Number of items	Mean	Standard deviation	Minimum–maximum	Cronbach's alpha	*n* **
Yards and entrances	8	2.5	0.58	1.00–4.00	0.81	296
Architecture	9	2.9	0.57	1.00–4.00	0.88	446
Acoustics	8	2.7	0.70	1.00–4.00	0.88	358
Lighting	6	3.0	0.59	1.00–4.00	0.73	319
Durability	7	2.8	0.66	1.00–4.00	0.89	227
Functionality	6	2.5	0.71	1.00–4.00	0.88	406
Indoor conditions	8	2.4	0.57	1.00–4.00	0.70	353
Safety and security	19	3.2	0.45	1.21–4.00	0.92	197
Privacy	7	2.8	0.68	1.00–4.00	0.87	267
Social interaction	7	2.7	0.73	1.00–4.00	0.84	231
Comfort	17	2.6	0.51	1.18–4.00	0.89	296
Accessibility	8	3.2	0.55	1.25–4.00	0.91	197
Usability	6	2.7	0.60	1.00–4.00	0.82	297
Postoccupancy evaluation*	116	2.8	0.43	1.16–3.98	0.99	52
Work engagement	3	4.4	0.99	0.00–6.00	0.80	510
Turnover intention	3	2.3	1.35	1.00–6.00	0.90	510

* Whole scale (sum variable).

** *n* Values used in the Cronbach's alpha calculations.

### Satisfaction With the Physical Work Environment in a New Hospital

The mean from the overall POE among all respondents was 2.8 (SD 0.43) (Table 2). All categories except indoor conditions achieved a mean of 2.5. The staff was most satisfied with safety and security (mean 3.2, SD 0.45), accessibility (mean 3.2, SD 0.55), lighting (mean 3.0, SD 0.59), and architecture (mean 2.9, SD 0.57), and least satisfied with the indoor conditions (mean 2.4, SD 0.57). The categories with the second lowest means were yards and entrances (mean 2.5, SD 0.58) and functionality (mean 2.5, SD 0.71), although they both met the mean.

Examining the correlations indicated that the POE categories were either moderately or strongly correlated (Table 3). A weak-level correlation was found only between functionality and lighting. The categories that were most frequently strongly correlated with other POE categories were satisfaction with architecture, safety and security, and comfort. Satisfaction with the comfort of the PWE correlated strongly with 10 of the 12 other categories. Satisfaction with comfort was correlated with yards and entrances, architecture, acoustics, lighting, functionality, indoor conditions, safety and security, social interaction, accessibility, and usability (*r* = 0.52–0.64, *p* < .001). Satisfaction with the architecture of the building showed strong correlations with satisfaction with yards and entrances, acoustics, durability, functionality, safety and security, comfort, accessibility, and usability (*r* = 0.52–0.59, *p* < .001). Satisfaction with safety and security was strongly correlated with comfort, accessibility, architecture, acoustics, lighting, durability, functionality, indoor conditions, and usability (*r* = 0.51–0.72, *p* < .001).

**Table 3.** Correlations of Study Variables.

Study variables	1	2	3	4	5	6	7	8	9	10	11	12	13	14	15
1. Yards and entrances	1														
2. Architecture	0.57	1													
3. Acoustics	0.40	0.52	1												
4. Lighting	0.38	0.47	0.49	1											
5. Durability	0.38	0.56	0.42	0.44	1										
6. Functionality	0.45	0.58	0.46	0.26	0.44	1									
7. Indoor conditions	0.39	0.49	0.49	0.50	0.42	0.50	1								
8. Safety and security	0.42	0.58	0.51	0.59	0.56	0.52	0.51	1							
9. Privacy	0.35	0.40	0.41	0.31	0.32	0.43	0.33	0.46	1						
10. Social interaction	0.46	0.40	0.40	0.40	0.33	0.44	0.38	0.42	0.48	1					
11. Comfort	0.52	0.57	0.52	0.60	0,48	0.61	0.58	0.58	0.46	0.52	1				
12. Accessibility	0.41	0.55	0.44	0.52	0.41	0.43	0.45	0.72	0.45	0.44	0.57	1			
13. Usability	0.47	0.59	0.46	0.54	0.47	0.66	0.49	0.61	0.37	0.43	0.64	0.55	1		
14. Work engagement	0.24	0.30	0.28	0.24	0.25	0.26	0.27	0.33	0.21	0.21	0.35	0.28	0.30	1	
15. Turnover intention	−0.17	−0.19	−0.20	−0.24	−0.16	−0.22	−0.21	−0.23	−0.11	−0.20	−0.29	−0.20	−0.25	−0.48	1
Post-occupancy evaluation														0.37	−0.28

Spearman's rho correlation coefficient for all variables *p* < .001.

### The Relationship Between Background Factors and Satisfaction in the Physical Work Environment

In the multifactor analysis of variance, the physicians were more satisfied with the physical work environment than nurses (mean 3.0 vs. 2.7, *p* < .001; Table 4). The satisfaction level with the PWE was also higher among hospital support and logistics staff compared to nurses (mean 3.1 vs. 2.7, *p* = .017). Physicians expressed greater satisfaction with the physical work environment compared to nurses with the hospital's architecture (mean 3.3 vs. 2.9, *p* < .001), yards and entrances (mean 2.8 vs. 2.5, *p* < .011), lighting (mean 3.4 vs. 3.0, *p* < .001), functionality (mean 2.9 vs. 2.4, *p* = .001), indoor conditions (mean 2.8 vs. 2.4, *p* = .002), safety and security (mean 3.3 vs. 3.1, *p* = .049), social interaction (mean 3.0 vs. 2.6, *p* = .023), comfort (mean 2.8 vs. 2.5, *p* = .003), and usability (mean 3.0 vs. 2.6, *p* = .008). Hospital support and logistics staff were more satisfied than nurses with the functionality (mean 3.0 vs. 2.4, *p* = .025), privacy (mean 3.1 vs. 2.5, *p* = .018), comfort (mean 3.1 vs. 2.5, *p* < .001), and usability (mean 3.2 vs. 2.6, *p* = .008). No statistically significant differences existed between professional statuses in acoustics, durability, and accessibility.

**Table 4.** The Relationship Between Professional Status, the Categories of Postoccupancy Evaluation, Work Engagement, and Turnover Intention.

	Professional status and pairwise comparisons									
	Nurses		Physicians		Other healthcare professionals		Nonclinical staff		Hospital support and logistics staff	
Category^a^	*n*	Mean, *p*-value	*N*	Mean, *p*-value	*N*	Mean, *p*-value	*N*	Mean, *p*-value	*n*	Mean, *p*-value
Yards and entrances	277	2.5 (*p* = .011)	61	2.8*	70	2.5	53	2.6	42	2.9
Architecture	276	2.9 (*p* < .001)	62	3.3*	71	2.9 (*p* = .020)	53	3.0 (*p* = .027)	42	3.1
Lighting	276	3.0 (*p* < .001)	61	3.4*	71	3.2	51	3.3	42	3.4
Functionality	276	2.4*	60	2.9 (*p* = .001)	71	2.5	51	2.5	41	3.0 (*p* = .025)
Indoor conditions	277	2.4 (*p* = .002)	61	2.8*	71	2.5	53	2.6	42	2.6
Safety and security	277	3.1 (*p* = .049)	62	3.3*	71	3.1	53	3.3	42	3.3
Privacy	268	2.5 (*p* = .018)	50	2.8	57	2.6	41	2.6	24	3.1*
Social interaction	252	2.6 (*p* = .023)	46	3.0*	58	2.7	40	2.9	34	3.1
Comfort Comparison x–y	277	2.5 (*p* = .003)	61	2.8*	71	2.7	53	2.8	42	3.1
Comparison x–y	277	2.5 (*p* < .001)			71	2.7 (*p* = .050)			42	3.1*
Comparison x–y	277	2.5 (*p* = .044)					53	2.8*		
Usability Comparison x–y	274	2.6 (*p* = .002)	59	3.0*	69	2.7	52	2.7	42	3.2
Comparison x–y	274	2.6 (*p* = .008)			69	2.7 (*p* = .030)			42	3.2*
Postoccupancy evaluation	277	2.7 (*p* < .001)	62	3.0*	71	2.8	53	2.9	42	3.1
Comparison x–y	277	2.7 (*p* = .017)							42	3.1*
Turnover intention	277	2.1 (*p* < .001)	62	1.2*	71	2.0 (*p* = .039)	53	2.0 (*p* = .038)	42	2.1

Category^b^	n	Mean	n	Mean	n	Mean	n	Mean	n	Mean
Acoustics	281	2.7	63	2.9	71	2.6	51	2.5	41	3.0
Durability	227	2.7	44	3.1	60	2.9	44	2.8	42	2.6
Accessibility	276	3.1	51	3.4	59	3.2	47	3.3	41	3.3
Work engagement	281	4.2	63	4.7	71	4.6	53	4.7	42	4.5

^a^ Multifactor analysis of variance (*object of pairwise comparison).

^b^ Descriptive statistics (no statistically significant pairwise differences in pairwise comparisons).

The highest “does not concern me” responses were for durability (27%–53%), the patient's social interaction (20%–42%), and privacy (16%–40%). Compared to other professional groups, hospital support and logistics staff responded proportionally more to statements regarding durability and less to statements regarding privacy.

Age was associated with the perception of patient privacy fulfillment. Younger employees perceived patient privacy to be better than older employees. One year of age reduced the mean of the sum variable by 0.012. Age was not associated with satisfaction for other categories of the physical work environment.

### The Relationship Between Satisfaction With the Physical Work Environment, Work Engagement, and Turnover Intention

Among all respondents, work engagement was perceived as moderate (mean 4.4; Table 2), with nurses (mean 4.2) and hospital support and logistics staff (mean 4.5) reporting moderate levels, whereas physicians (mean 4.7), nonclinical staff (mean 4.7), and other healthcare staff (mean 4.6) reported above-average levels of work engagement (Table 3). The POE showed a moderate correlation with work engagement (*r* = 0.37, *p* < .001; Table 4). The experience of the physical work environment's comfort (*r* = 0.35, *p* < .001) and satisfaction with security and safety (*r* = 0.33, *p* < .001) had a moderate correlation with work engagement (Table 3). Other POE categories had a weak correlation with work engagement. A weak correlation was observed between satisfaction with the physical work environment and turnover intention.

## Discussion

Our study produced novel information about the relationship between satisfaction with the physical work environment, work engagement, and turnover intention among staff in a new hospital setting. Results show that overall satisfaction with the physical work environment in the new hospital is relatively high, above the desired mean. A study by Jouppila (2022) using the same POE questionnaire in a new hospital showed similar results in all categories except social interaction, which scored higher in this study. The timing of the POE in our study may have contributed to the overall higher satisfaction levels, as it was conducted nearly 2 years after relocation. Pruijsten et al. (2024) found that nurses’ perceptions of the physical environment were less favorable during the first year postrelocation, and notable improvements were seen after 2 years. This suggests that conducting a POE after habituation to the new physical work environment may provide a more accurate reflection of the new hospital and its influence on staff wellbeing.

According to our results, the more satisfied the new hospital staff were with safety and security, comfort, and architecture, the more satisfied they were with other aspects of the physical work environment. In our results, staff were least satisfied with the indoor conditions.

This study showed differences between professional groups’ satisfaction with the physical work environment. Physicians and hospital support and logistics staff reported higher satisfaction levels with the physical work environment than other groups, with physicians being the most satisfied. Nurses were the least satisfied with the physical work environment in nearly all categories. The study brought new information about the possible role of the profession, as research on staff concerning the variation of health and comfort aspects across different departments has been limited (Eijkelenboom & Bluyssen, 2022). Age was associated with perceptions of satisfaction in patient privacy, with younger employees being more satisfied.

A generally moderate positive correlation was found between satisfaction with the physical work environment and work engagement, meaning that satisfaction with the physical work environment was associated with higher work engagement. Satisfaction with safety and security, and comfort also showed moderate correlations with work engagement. The connection between different elements of the physical environment and work engagement or other categories of the physical environment had not emerged in earlier research, making this a surprising result.

Physicians were most satisfied with the physical work environment, but also had the highest work engagement. It can be assumed that they are not having an increased risk of burnout (Wang et al., 2023). High satisfaction with the physical work environment has been shown to have a positive impact on reducing burnout rates among physicians (Bentulila et al., 2024). On the contrary, a poor physical work environment has been shown to generate high levels of stress and burnout in healthcare workers (Lupo et al., 2021). It is worth noting that nurses in our study were the least satisfied with the physical work environment and had the lowest levels of work engagement. Additionally, the findings revealed an association between satisfaction with the physical work environment and higher work engagement. Considering the global workforce shortage (WHO, 2022b, 2023) and given that nurses are the largest professional group in hospitals, hospital organizations and planners of new facilities need to address the factors contributing to dissatisfaction in the physical work environment.

In this study, satisfaction with the physical work environment does not seem to impact turnover intention. This may be due to the successful design of the hospital, as overall satisfaction with the physical work environment was above the preferred mean. While our findings did not indicate a direct link between satisfaction with the physical work environment and turnover intention, one previous study has shown connections between the physical work environment and staff retention as part of the broader economic rationale for investing in hospital infrastructure. For example, enhanced design features have improved staff satisfaction and reduced turnover, ultimately contributing to long-term operational cost savings (Sadler et al., 2011). Previous studies have also shown that job satisfaction is associated with work engagement, and it is a well-known protective factor against turnover intention (Fasbender et al., 2019; Hu et al., 2022; Lu et al., 2019; Schaufeli et al., 2019; Wang et al., 2023). Job satisfaction has also shown a strong relationship with job stress, pay and benefits, administrative support, and perceived organizational support (Lu et al., 2019).

## Limitations

This study has some limitations. The outcomes may not apply universally to other new hospitals due to the particular social environment and patient demographics of this facility. Our study focused on a Finnish public hospital, which restricted the geographical and cultural scope. The use of convenience sampling may impact the generalizability of the results, and the study's outcomes may represent selection bias because involvement was not mandatory. Although precise internal data on staff group distributions were unavailable due to data protection policies, the respondent profile broadly aligned with the hospital's staff composition based on survey background variables. Employing random sampling might have improved generalizability (Polit & Beck, 2022) by ensuring a more representative sample of the hospital staff. The external validity of the study may be limited by the relatively low response rate (33%), which could be affected by factors such as excessive workloads, limited time for participation, and ongoing organizational changes due to the recent healthcare and social reform in Finland, and the length of the comprehensive 116-item POE questionnaire.

Our study utilized self-reported measures, which may have led to social desirability response bias. The responses to the online questionnaire were quite positive, which can indicate a selection bias. Staff members with high turnover intention or low work engagement may not have answered the questionnaire. Also, there were relatively few men in the sample, <10%, and their low representation may have influenced the results, though the proportion of all males in the hospital remains unknown.

## Conclusions

This study highlights the importance of creating a well-functioning work environment in healthcare settings, noting that satisfaction with the physical work environment can vary among different professional groups. The results suggest that when planning new hospitals, attention should be paid to developing the physical work environment, especially in terms of safety, security, and comfort, since it may impact the staff's work engagement. Satisfaction with the physical work environment does not appear to affect staff turnover intention.

More research is required to explore the effects of old hospital buildings on staff, particularly in terms of how factors like poor indoor air quality may influence turnover intention. The results might differ from studies conducted in a new, modern hospital like this one. Presumably, an old, dysfunctional work environment could have a more significant negative impact on work engagement and turnover intention. Additionally, the challenges for future research include studying the impact of the physical work environment on work engagement and turnover intention in the context of new hospital projects, using both pre- and postsurveys among staff. This approach would contribute to more user-centered design and enable a better understanding of the relationship between the physical work environment and work engagement and turnover intention. Future research should also explore what causes nurses’ dissatisfaction in the physical work environment, especially since they are the largest professional group, as addressing these issues is vital for enhancing their work engagement. This study is only based on a staff questionnaire, excluding the perspectives of patients and their families. Therefore, we suggest that future POEs incorporate patient perspectives, as their experiences are essential for assessing the effectiveness of new hospital designs. It is also important to recognize that the experiences of patients and their families are crucial in evaluating the success of new hospital designs. Additionally, a challenge for future research would be to explore using adaptive or modular surveys to reduce participant burden while maintaining comprehensive data collection.

## Implications for Practice

- When designing a new hospital, attention should be paid to safety, security and comfort. Hospital designers should prioritise these elements in new hospital projects to foster work engagement, which supports job satisfaction, performance, and retention.
- Understanding and addressing potential differences between professional groups is fundamental to improving work engagement. Since nurses reported the lowest satisfaction with the physical work environment and the lowest work engagement, design interventions should specifically address their needs, as nurses are usually the largest professional group in hospitals. Practical steps include involving nurses early in the design process to ensure their needs are met in the planning phase.
- Since satisfaction with comfort and safety had the strongest associations with work engagement, these elements should not be considered optional design luxuries but as strategic investments in workforce well-being. Enhancing these features can support staff engagement, which is especially critical in the global healthcare workforce shortage.

## Supplemental Material

sj-docx-1-her-10.1177_19375867251351026 - Supplemental material for Postoccupancy Evaluation of a New Hospital: The Relationship With Work Engagement Supplemental material, sj-docx-1-her-10.1177_19375867251351026 for Postoccupancy Evaluation of a New Hospital: The Relationship With Work Engagement by Hanna Petäjä, Pinja Krook, Suvi Kuha, Jouko Katajisto and Outi Kanste in HERD: Health Environments Research & Design Journal
